# Applications of Self-Driving Vehicles in an Aging Population

**DOI:** 10.2196/66180

**Published:** 2025-04-28

**Authors:** Sara Shu, Benjamin K P Woo

**Affiliations:** 1 Department of Community Internal Medicine, Geriatrics and Palliative, Mayo Clinic, 200 First Street SW, Rochester, MN, 55901, United States, 1 5072845278; 2 University of California, Los Angeles, Los Angeles, CA, United States; 3 Asian American Studies Center, University of California, Los Angeles, Los Angeles, CA, United States; 4 Chinese American Health Promotion Laboratory, University of California, Los Angeles, Los Angeles, CA, United States

**Keywords:** self-driving, driverless, driver, autonomous vehicles, car, transportation, mobility, travel, vehicle, driving, artificial intelligence, gerontology, geriatric, older, elderly, aging, healthy aging, older adult, autonomy, independence, aging in place, health equity

## Abstract

The proportion of older adult drivers is increasing and represents a growing population that must contemplate reducing driving and eventually stopping driving. The advent of self-driving vehicles opens vast possibilities with practical and far-reaching applications for our aging population. Advancing technologies in transportation may help to overcome transportation barriers for less mobile individuals, transcend social and geographical isolation, and improve resource and medical access. Herein, we propose various applications and benefits that self-driving vehicles have in maintaining independence and autonomy specifically for our aging population to preserve aging.

## Introduction

In the United States, driving remains a symbol of independence and is an important means to maintain a connection to and identity within the community. For many community-dwelling older adults, private vehicles remain the primary mode of transportation. In the United States, there are over 55 million licensed automobile drivers over 65 years of age, comprising over 25% of the total driving population [1]. Motor vehicle collisions are among the most common causes of nonfatal and fatal injuries in the United States in older adults [2,3]. Older adults have higher mortality compared to a younger population with similar motor vehicle collision-related injuries [4]. The association between increased dangers of driving and collisions in the older adult population is well-known. With increasing age comes impairments in vision and visuospatial and reaction times, all of which make it more dangerous and difficult to navigate safely [5]. Primary care providers and associated interdisciplinary teams are in a unique position to assess the fitness of older adults to drive safely and provide alternative transportation recommendations [6,7]. Developments in technology may also provide tools to assist with the assessment of driving safety. For example, speech data analysis during interactions with voice assistants has been suggested to accurately predict future accident experiences in older drivers [8].

Environmental factors such as congestion of roads and the distance to the locations of goods and services can influence driving reduction and cessation in older adults [9]. Even medical events requiring hospitalization have been shown to correlate with immediate or eventual driving reduction or cessation, which ultimately compromises the autonomy of individuals [10]. Moreover, driving cessation has been associated with declines in general health and mental, physical, social, and cognitive function, and has even been associated with increased risk of admission to long-term care facilities and mortality [11,12]. In this paper, we aim to provide an updated review of current and proposed applications of self-driving vehicles, particularly through the integration and application of mobility as a service to assist with prolonging the autonomy of aging persons, as well as review the limitations and future directions that have yet to be explored.

## Towards Age-Friendly Communities

Over the past several decades, there has been a push to develop communities and city infrastructure to be more supportive of an aging population in areas such as housing, transportation, and access to health care and social services. There is great heterogeneity in the driving challenges and concerns of older adult drivers in different geographical settings: urban, suburban, and rural [13]. Differences in community infrastructure have shown varying densities in the incidence of fatal crashes for older drivers. For example, the majority of fatal crashes occurred on rural roads, while suburban and urban roads conferred a different likelihood of crash location and mechanisms, such as multivehicle crashes, as well as those within intersections versus multilane roads [14]. The movement towards age-friendly communities has driven the development of the World Health Organization Global Network for Age-Friendly Cities and Communities to provide a framework of considerations when developing age-friendly environments [15]. These include the development of technologies, services, and systems to transcend physical and social barriers that can promote the inclusion and continued integration and engagement of older adults in the community. As there are increasing needs in the rapidly growing older adult population, there will be an increased need to develop innovative strategies to meet them.

Self-driving vehicles hold promise in maintaining autonomy and maintaining independence to allow living in and remaining at home for as long as possible. When the option of driving autonomy is exhausted, one begins to recruit assistance from social networks comprising relatives, neighbors, and friends. However, as social networks organically decrease among an aging population, socially isolated individuals may not have the luxury to call on such assistance. The reliance on public transportation as a primary mode of transportation is not always feasible nor uniformly available across the United States. Extreme temperatures and climates during the winter and summer months may furthermore be a barrier for older adults to use public transportation. Volunteer transportation programs, senior services, or service operations that charge a small fee are available but are met with similar limitations in access depending on the local infrastructure and individual socioeconomic status. Therefore, finding a reliable and affordable means of transportation remains a great challenge in our aging society.

## Applications of Self-Driving Vehicles

Self-driving (also known as autonomous) vehicles present a promising alternative and emerging transportation option for our older adult population [16,17]. Semiautonomous vehicles already have applications for use as cognitive-assistive devices for community-dwelling older adults owing to features such as onboard navigation, parking assistance, lane departure, and collision avoidance technologies [18]. As fully autonomous vehicle technology continues to develop, the applications are far reaching. By freeing the need to engage another community-dwelling adult or one in the social network, the availability of transportation options will inadvertently increase. In metropolitan areas where self-driving vehicles have been launched as mobility services, many individuals are already using these mobility services for commuting to and from work. The use of this technology in the older population can help to close the divide in populations otherwise socially isolated and enable them to once again be independent through their community and be able to engage in the community-based or group activities they once enjoyed. It would significantly relieve the caregiver’s burden of having to provide transportation to and from medical appointments, the grocery store, barber shop, or other social functions and leisure activities. Similar mobility service efforts are seen in the European Union and Asia, where working towards cooperative, connected, and automated mobility is being optimized to embolden digital connectivity between vehicles, the transportation infrastructure, and other road users. These so-called cooperative intelligent transport systems are the foundation for the widespread use and applications of these technologies to provide services for otherwise homebound community-dwelling older adults with limitations that would preclude them from independent and safe utilization of their own motor vehicles.

As the market is already saturated with home food and medical supply delivery services, self-driving vehicles can eventually be equipped to undertake these tasks, thereby relieving the current workforce to engage in other areas of need. As much of the home delivery services rely on assistance from community-dwelling adults, these individuals would now have increased caregiving capacities for their aging community and loved ones, to serve in capacities where technology is unable to provide assistance. Some of the country’s largest ride-share companies are already pioneering the driverless vehicle space by partnering with home-based care agencies [19]. By increasing the capacity and affordability of these services, the use of these technologies can help to provide solutions to building more equitable age-friendly, disability-friendly communities.

Similar advantages of self-driving vehicles can be extended to mobility-isolated persons or those with disabilities. In order to optimize accessibility to these populations, car designs should be optimized to be age-friendly and wheelchair accessible, such as being equipped with a ramp or capacity for the vehicle to lower itself to the curbside. Vehicles such as Toyota Sienna’s “Autono-MaaS” (Autonomous-Mobility as a Service) vehicles are already incorporating these features in partnership with transportation companies to provide increased transportation options for those with disabilities. Companies such as May Mobility and Via have launched new and free self-driving shuttle services called “Accessibili-D” for the local inhabitants of Detroit, Michigan aged 65 years and older or those with disabilities [20]. These on-demand shuttle services have deployed similar services in Texas, Arizona, and throughout Michigan and have expanded into retirement communities. The “goMARTI” (Minnesota’s Autonomous Rural Transit Initiative) in Grand Rapids, Minnesota, was the first pilot to provide free, on-demand rides with the American Disabilities Act (ADA)-compliant vehicles to improve mobility to those otherwise mobility-isolated in rural areas and during inclement weather [21].

With the continued development of artificial intelligence (AI) technology, medical capabilities could eventually be installed in autonomous vehicles to be able to detect and respond to medical emergencies. They could be equipped with emergency medical supplies in addition to sensors and technology that could detect the need to redirect to the nearest medical center, connect with a remote telemedicine provider, or provide basic equipment such as oxygen or epinephrine pens when indicated. Vehicles could even be engineered to detect mood fluctuations including depressed, anxious, or agitated states, and be equipped with robotic animal interventions for temporal therapy or connect individuals to telemedicine assistance or crisis response teams. These so-called autonomous mobile clinics equipped with AI software, medical diagnostic detection, equipment, and telemedicine capabilities could reshape and facilitate prompt medical care and can even help to bring medical care to once-difficult-to-reach, more rural populations [22,23]. Epidemiologically conscious uses of self-driving vehicles have already been piloted in the days of the COVID-19 pandemic, such as contactless goods transportation and transporting patients in isolation to prevent exposure to other high-infection risks. Nevertheless, such widespread integration and the adoption of clinical applications of AI have yet to be subject to thorough clinical validation and approval by the appropriate regulatory authorities [24].

## Challenges and Limitations

The limitations of self-driving vehicles are many, and as far as our imaginations can carry us considering the tremendous potential these technologies have to offer, so too do foreseen issues and concerns arise. Hypothetical issues of the unknown have guided various expert opinions. Driving principles in regard to road user safety are expected to be upheld; however, there are countless scenarios by which these self-driving vehicles must navigate tensions between obeying the traffic code and avoiding human harm. This highlights the importance of the translation of these frameworks into the engineering of such self-driving vehicles to guarantee consistency and safety in behavior that is driven by automated technology [25]. Moreover, in addition to predicting the unpredictability of road interactions, passenger safety is needed as well. For example, in caring for an older adult with dementia, one with limited mobility, or one with mental health concerns, it is important to have the assurance of safety for the otherwise vulnerable adult, such as precluding drop-off into an unfavorable environment. For example, a person with compromised cognitive abilities may not recognize that it may be unsafe to be outside for extended periods of time in a heatwave, or a person with impulsivity may not comprehend the dangers of going to a casino. Such ethical dilemmas tread the fine line between autonomy and paternalism, especially when such technology works to expand the independence and autonomy of an increasingly diverse, vulnerable population. Whether autonomous vehicles should have the ability to override the decisions of their passengers under the pretense of responsibility for safety over vulnerable populations or risk the repercussions for invading the right to autonomy has yet to be explored.

The implications of AI software on passenger privacy in autonomous vehicles will always be a concern. As the software of these autonomous vehicles could potentially be targets of cyberattacks, the kind of protections that would be placed on ensuring the safety of its passengers is important. Moreover, the use of these technologies would result in varying perceptions and attitudes from different potential users, all of which are also important. Various small-scale studies have captured such perceptions in the older adult population, including in persons with dementia [26-31]. It is important, therefore, to address targeted user populations’ concerns about the cost, functionality, performance, and trustworthiness of the automated vehicles to suit their needs. Social determinants of health can also affect the accessibility of these technologies and have the potential to further marginalize underserved communities and populations; therefore, affordability needs to be considered. By partnering with local, state, and national departments of transportation and agencies on aging, solutions are needed for the promotion of equity, accessibility, and inclusion of all the mobility needs of older adults as well as individuals with disabilities and caregivers, regardless of the socioeconomic status.

## Conclusion

In summary, the advent of self-driving, or autonomous, vehicles can have beneficial effects on how individuals are able to age while ensuring autonomy. We are presently at a unique crossroads of personalizing this developing technology to facilitate and optimize independence for the older adult population and maintain autonomy for as long as possible. Engaging the proper stakeholders may enable the rapid development of these technologies to promote a more age-friendly society.

## Abbreviations

ADA: American Disabilities Act
AI: artificial intelligence

## References

[1] Federal Highway Administration, Department of Transportation (US) Distribution of licensed drivers 2022. https://www.fhwa.dot.gov/policyinformation/statistics/2022/.

[2] Platts-Mills TF, Hunold KM, Esserman DA, Sloane PD, McLean SA (2012). Motor vehicle collision-related emergency department visits by older adults in the United States. Acad Emerg Med.

[3] Dellinger AM, Stevens JA (2006). The injury problem among older adults: mortality, morbidity and costs. J Safety Res.

[4] Perdue PW, Watts DD, Kaufmann CR, Trask AL (1998). Differences in mortality between elderly and younger adult trauma patients: geriatric status increases risk of delayed death. J Trauma.

[5] Anstey KJ, Windsor TD, Luszcz MA, Andrews GR (2006). Predicting driving cessation over 5 years in older adults: psychological well-being and cognitive competence are stronger predictors than physical health. J Am Geriatr Soc.

[6] Pomidor A (2019). Clinician’s Guide to Assessing and Counseling Older Drivers.

[7] Hill LJN, Pignolo RJ, Tung EE (2019). Assessing and counseling the older driver: a concise review for the generalist clinician. Mayo Clin Proc.

[8] Yamada Y, Shinkawa K, Kobayashi M (2021). Using speech data from interactions with a voice assistant to predict the risk of future accidents for older drivers: prospective cohort study. J Med Internet Res.

[9] Vivoda JM, Heeringa SG, Schulz AJ, Grengs J, Connell CM (2017). The influence of the transportation environment on driving reduction and cessation. Gerontologist.

[10] Gaulton TG, Neuman MD, Brown RT, Betz ME (2021). Association of hospitalization with driving reduction and cessation in older adults. J Am Geriatr Soc.

[11] Chihuri S, Mielenz TJ, DiMaggio CJ (2016). Driving cessation and health outcomes in older adults. J Am Geriatr Soc.

[12] Qin W, Xiang X, Taylor H (2020). Driving cessation and social isolation in older adults. J Aging Health.

[13] Payyanadan RP, Lee JD, Grepo LC (2018). Challenges for older drivers in urban, suburban, and rural settings. Geriatrics (Basel).

[14] Zwerling C, Peek-Asa C, Whitten PS, Choi SW, Sprince NL, Jones MP (2005). Fatal motor vehicle crashes in rural and urban areas: decomposing rates into contributing factors. Inj Prev.

[15] (2018). The global network for age-friendly cities and communities: looking back over the last decade,looking forward to the next. https://www.who.int/publications/i/item/WHO-FWC-ALC-18.4.

[16] Abdi S, de Witte L, Hawley M (2020). Emerging technologies with potential care and support applications for older people: review of gray literature. JMIR Aging.

[17] Shu S, Woo BKP (2021). Use of technology and social media in dementia care: current and future directions. World J Psychiatry.

[18] Knoefel F, Wallace B, Goubran R, Sabra I, Marshall S (2019). Semi-autonomous vehicles as a cognitive assistive device for older adults. Geriatrics (Basel).

[19] Donlan A, Home Health Care News (2020). Driverless cars finding new lane in home-based care. https://homehealthcarenews.com/2020/03/driverless-cars-finding-new-lane-in-home-based-care/.

[20] Hope G, IOT World Today (2024). Self-driving shuttle for elderly, disabled residents launches in detroit. https://www.iotworldtoday.com/transportation-logistics/self-driving-shuttle-for-elderly-disabled-residents-launches-in-detroit.

[21] Bellan R, Tech Crunch (2022). May mobility, via launch wheelchair-accessible autonomous shuttle service. https://techcrunch.com/2022/09/28/may-mobility-via-launch-wheelchair-accessible-autonomous-shuttle-service.

[22] Liu S, Kong A, Huang Y, Liu X (2022). Autonomous mobile clinics. Bull World Health Organ.

[23] Eliot L, Forbes (2020). The problems of medical emergencies while inside a self-driving car are going to be challenging. https://www.forbes.com/sites/lanceeliot/2020/03/17/the-problems-of-medical-emergencies-while-inside-a-self-driving-car-are-going-to-be-challenging/?sh=5179515a1670.

[24] van Hartskamp M, Consoli S, Verhaegh W, Petkovic M, van de Stolpe A (2019). Artificial intelligence in clinical health care applications: viewpoint. Interact J Med Res.

[25] D’Amato A, Dancel S, Pilutti J (2022). Exceptional driving principles for autonomous vehicles. J of Law and Mobility.

[26] Haghzare S, Stasiulis E, Delfi G (2023). Automated vehicles for people with dementia: a “tremendous potential” that “has ways to go”-reports of a qualitative study. Gerontologist.

[27] Doulabi S, Hassan HM, Li B (2023). Senior Americans’ perceptions, attitudes, and safety concerns toward autonomous vehicles (AVs). J Safety Res.

[28] Oxley J, Charlton J, Logan D, O’Hern S, Koppel S, Meuleners L (2019). Safer vehicles and technology for older adults. Traffic Inj Prev.

[29] Faverio M, Pew Research Center (2022). Older americans more wary than younger adults about prospect of driverless cars on the road. https://www.pewresearch.org/short-reads/2022/10/24/older-americans-more-wary-than-younger-adults-about-prospect-of-driverless-cars-on-the-road/.

[30] Zandieh R, Acheampong RA (2021). Mobility and healthy ageing in the city: Exploring opportunities and challenges of autonomous vehicles for older adults’ outdoor mobility. Cities.

[31] Lajunen T, Sullman MJM (2021). Attitudes toward four levels of self-driving technology among elderly drivers. Front Psychol.

